# Acquired Abdominal Intercostal Hernia: A Case Report and Literature Review

**DOI:** 10.1155/2014/456053

**Published:** 2014-08-17

**Authors:** Salim Abunnaja, Kevin Chysna, Inam Shaikh, Giuseppe Tripodi

**Affiliations:** The Stanley Dudrick Department of Surgery, Saint Mary's Hospital, 56 Franklin Street, Waterbury, CT 06706, USA

## Abstract

Acquired abdominal intercostal hernia (AAIH) is a rare disease phenomenon where intra-abdominal contents reach the intercostal space directly from the peritoneal cavity through an acquired defect in the abdominal wall musculature and fascia. We discuss a case of a 51-year-old obese female who arrived to the emergency room with a painful swelling between her left 10th rib and 11th rib. She gave a history of a stab wound to the area 15 years earlier. A CT scan revealed a fat containing intercostal hernia with no diaphragmatic defect. An open operative approach with a hernia patch was used to repair this hernia. These hernias are difficult to diagnose, so a high clinical suspicion and thorough history and physical exam are important. This review discusses pathogenesis, clinical presentation, complications, and appropriate treatment strategies of AAIH.

## 1. Introduction

Intercostal hernias are rare phenomena caused by a disruption or weakness in the thoracoabdominal wall musculature resulting in herniation of fascia layers between adjacent ribs. Historically, these hernias have been characterized by their contents. They may only be an empty sac comprised solely of fascia elements [1] or may contain abdominal and thoracic viscera, such as liver [2], lung [3], small and large bowel [4, 5], omentum (present case), or gallbladder [6]. Intercostal hernias have also been categorized on the basis of their etiology, with majority resulting from trauma (blunt injury [7], penetrating injury [8], rib fractures [9], or prior surgery [4]). Rarely, they occur spontaneously or with congenital syndromes [10–12]. Recently intercostal hernias have been divided into two types: those with a diaphragmatic defect and those without a diaphragmatic defect [9, 13]. Many authors, however, do not distinguish between the two [2, 9, 12, 14–16], as several cases labeled as intercostal hernias without diaphragmatic involvement had, upon careful examination, diaphragmatic defects [9, 12, 13, 17]. We, however, believe that the term “acquired abdominal intercostal hernia” (AAIH) could be reserved for cases in which the intra-abdominal contents reach the intercostal space directly from the peritoneal cavity through an acquired defect in the abdominal wall musculature or fascia [9, 13]. When viscera herniate through a diaphragmatic defect, the term “transdiaphragmatic intercostal hernia” (TIH) should be used. Since the two types may have overlapping but distinct clinical presentations, pose unique therapeutic challenges, and may require different surgical strategies, they should remain as separate pathologic entities.

## 2. Case Report

This is a fifty-one-year-old obese and hypertensive female who presented with a painful mass at her left upper abdominal quadrant and lower chest for about 24 hours. In addition to pain, she complained of nausea but denied vomiting or changes in bowel habits. She reported a history of stab injury to her left chest about fifteen years ago. She has had this mass for several years but had remained asymptomatic. Workup by her primary care in the past including a computed tomography (CT) scan concluded that the mass was most likely a lipoma.

**Figure 1:**
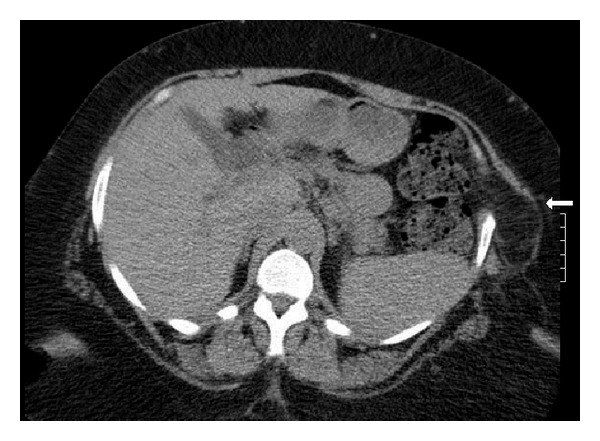
Axial CT view: intercostal hernia between 10th rib and 11th rib at left midaxillary line (white arrow).

**Figure 2:**
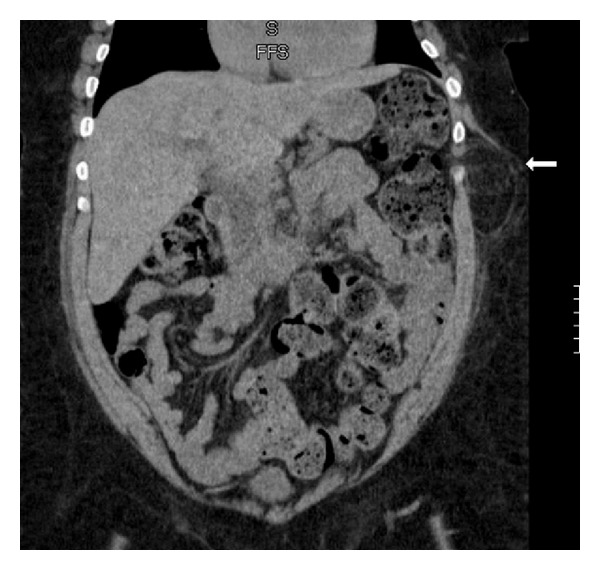
Coronal CT view: intercostal hernia between 10th rib and 11th rib at left midaxillary line (white arrow).

On physical examination, the patient was noted to be obese with a tender, firm, and nonreducible mass at the left upper quadrant and lower chest measuring about 8 × 8 cm. A new CT scan was obtained showing an abdominal intercostal hernia between the 11th rib and 10th rib. The hernia content was comprised of omentum, and no evidence of a diaphragmatic defect was seen on CT (Figures 1 and 2).

**Figure 3:**
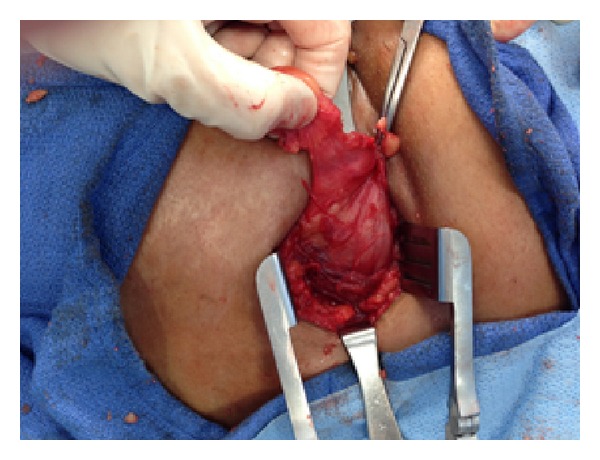
Exposure of the hernia sac.

**Figure 4:**
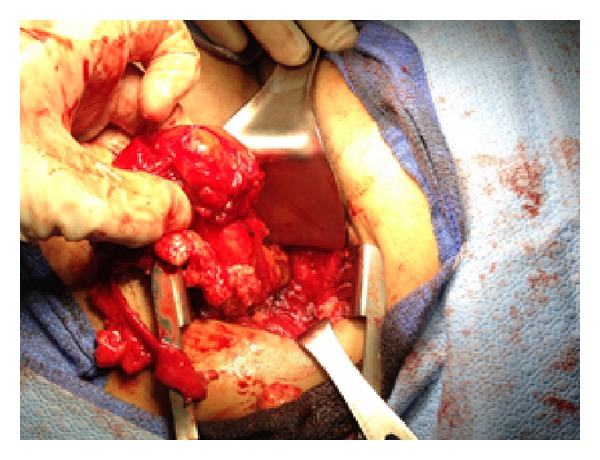
Dissection of the hernia sac of the surrounding subcutaneous tissue.

The patient was taken to the operative room where she was placed in a right lateral position. Under general anesthesia an incision was made over the hernia along the intercostal space. The hernia sac was identified and dissected clean of the surrounding subcutaneous tissue (Figures 3 and 4).

**Figure 5:**
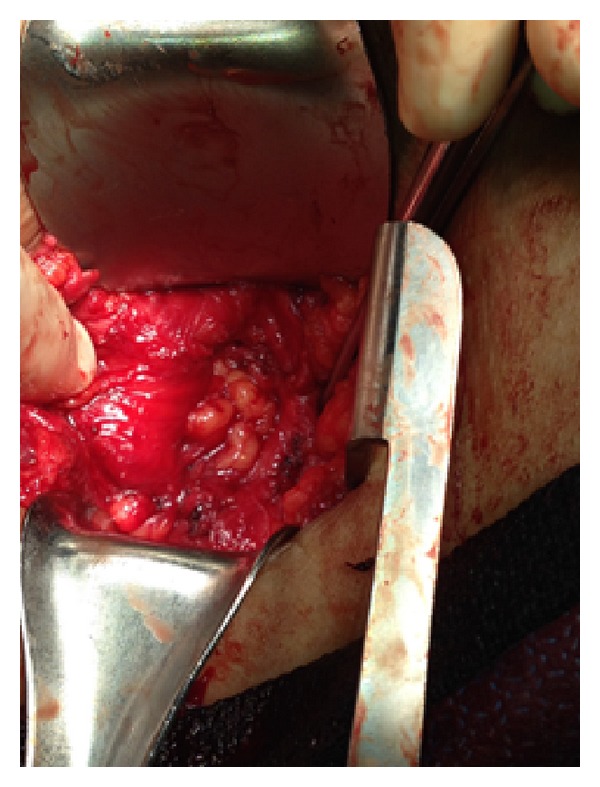
Exposure of defect.

The hernia sac was opened and found to contain omentum, which was reduced back into the peritoneal cavity. The sac was subsequently excised, exposing a clear defect between the tenth rib and eleventh rib (Figure 5).

**Figure 6:**
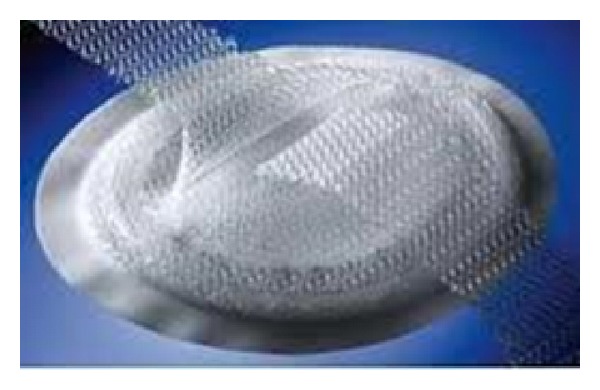
VENTRALEX Hernia Patch.

**Figure 7:**
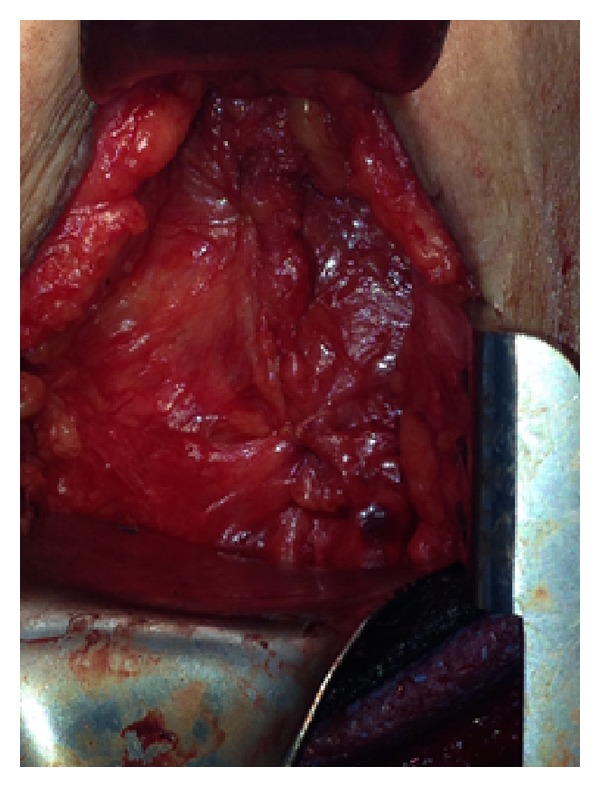
Approximation of muscle fascia on top of the patch.

A self-expanding polypropylene and ePTFE hernia patch (VENTRALEX Hernia Patch) (Figure 6) was then used to secure the defect, and the fascia of the intercostal and external oblique was approximated on top of the mesh using interrupted Vicryl stiches (Figure 7). The patient's postoperative course was uneventful and was discharged home on postoperative day two.

## 3. Discussion

Acquired abdominal intercostal hernia (AAIH) is an extremely rare phenomenon having only 19 cases reported in the literature worldwide [9]. By definition, AAIH does not involve a defect in the diaphragm, which, if present, is called transdiaphragmatic intercostal hernia (TIH). Our patient's hernia was previously reducible; however, at the time of presentation the hernia was incarcerated. The diagnosis of the AAIH was confirmed with computed topography (CT) scan and an open intercostal hernia repair with patch was performed.

Abdominal intercostal hernias (AIH) are due to weakened or torn muscular layers of the thoracoabdominal wall, which is unable to provide adequate resistance to the outward forces of visceral contents pressing against it during variations in internal pressure [18]. The outer layers of the hernia sac itself in AIH include the transthoracic fascia, transversalis fascia, and peritoneum [19] and may or may not contain contents from the peritoneum or thorax [9]. One mechanism causing tissue disruption, and accounting for 65% of all AAIH [9, 10, 20], is by major trauma: blunt forces, deceleration injuries, or penetrating injuries from sharp objects, like knives or fractured ribs [12]. Unlu et al. report several predisposing conditions to patients with intercostal hernias after minor traumatic events: COPD, asthma, diabetes mellitus, advanced age, treatment with steroids, excessive weight loss, and increased intra-abdominal pressure [12]. Such sudden or chronic increases in pressures may cause microtrauma to the fascia or muscles of thoracoabdominal wall [18]. Rib fractures can complicate the picture of AAIH because, in some instances, the jagged edges of the fractured ribs penetrate abdominal wall tissue, predisposing to a traumatic intercostal herniation [18, 22]. Other rare pathophysiological mechanisms that weaken the chest wall include congenital conditions decreasing tissue strength such as Ehlers-Danlos syndrome [23] and congenital conditions associated with chest wall defects like Poland syndrome  [11].

While disruption of the thoracoabdominal wall seems to be the only pathogenesis for the occurrence of abdominal intercostal hernias, it appears that it is not sufficient for all cases. It is likely that a combination of weakened tissues in the event of sudden increases in intra-abdominal pressure results in intercostal hernias or incarceration of previously reducible ones. This may explain why some patients with a distant history of anterior abdominal wall trauma, like in the case presented here, suddenly develop complications after years of being asymptomatic. The time interval between trauma and hospitalization for abdominal intercostal hernia, spontaneous or acquired, is highly variable. Some authors report hospitalization within the same day after trauma [24], while others report a 20 years span between trauma and hospitalization [25]. In the present case, the patient was hospitalized 15 years after a stab wound because of symptoms of pain and swelling that developed over the course of 24 hours. While it is not clear what triggered the sudden incarceration of the hernia and the subsequent symptoms in our patient, obesity was a notable risk factor. This case also emphasizes the importance of a thorough history, as this patient's stab wound 15 years ago helped support the diagnosis of AAIH.

Specific areas of the chest wall are more vulnerable to herniation than others due to inherent weakness in certain anatomical zones [18]. The chest wall is weak anteriorly from the costochondral junction to the sternum, as it lacks the support of the external intercostal muscle. Posteriorly, the internal intercostal muscles are absent from the costal angle to the vertebrae, contributing to another weak point [12, 26]. Interestingly, our patient's intercostal hernia did not occur around these areas of vulnerability but in a more reinforced area of the chest wall where all intercostal muscles reside. Most AAIH are located under the 9th rib without a preference to side, and main symptoms include chest swelling (85%) and pain or discomfort (76%) [9]. If bowel herniates, symptoms of obstruction may be present, with the most specific sign for this being the presence of bowel sounds in the chest   [19].

The diagnosis of any type of intercostal hernia can be difficult to make due to edema, hematoma, or obesity, which obscure the protruding abdominal wall contents [2]. For this reason, CT is the best diagnostic tool, since it not only provides excellent visualization but also offers a reliable means to establish a preoperative plan to repair the defect   [2].

Surgical management is necessary in nearly all cases due to risk of incarceration and strangulation of organs [27]. In fact, Erdas et al. report that 15% of AAIH are complicated by incarceration and strangulation of omentum, small and large bowel, or liver [9]. Other complications include a missed diaphragmatic tear or defect, which can predispose patients to recurrent intercostal hernias [9, 18]. Although deaths have not been reported in cases of AAIH, they have been reported in transdiaphragmatic intercostal hernias, mostly occurring as a consequence of hemorrhage from other associated injuries [18, 28]. Rarely, conservative management is warranted in elderly patients with multiple comorbidities who pose a high surgical risk. Conservative management has been reported in some asymptomatic patients [21, 27], but we recommend that nonsurgical measures in asymptomatic patients should only be undertaken after careful consideration of patient's age, risk of recurrence, acuteness of the hernia, comorbidities, surgical risk factors, and type and size of hernia.

Because there are so few reports of acquired abdominal intercostal hernias, determining the efficacy of various surgical techniques employed is difficult. The surgeon must account for many factors about the patient and the injury before deciding on a repair technique. Closure of the defect can be achieved by the direct approach, as in the present case, which consists of a thoracotomy (open intercostal incision) performed along the intercostal space. It can also be done by an indirect approach, which consists of laparoscopy or open abdominal incision (laparotomy) [2, 13, 26]. A combined open (direct) and laparoscopic (indirect) method was also successfully performed [27]. Techniques to repair the defect include primary closure, absorbable and nonabsorbable meshes and patches, and prosthetic mesh reinforced by cable banding [29].

In emergency situations, the open abdominal approach is the most prudent operative choice as it allows the surgeon easy access to other intra-abdominal injuries often associated with blunt or penetrating injuries to the abdomen and thorax [27]. Laparoscopic repair has also been performed in emergent settings where a visceral injury was present or could not be determined preoperatively [30]. Laparoscopy has its advantages, as it enables adequate management of compromised hernia contents, allows treatment of other intraperitoneal injuries, and is minimally invasive. However, its disadvantages make it less favorable than the open intercostal approach in noncomplicated cases [9]. Such disadvantages include a greater level of expertise required, the placement of the mesh intra-abdominally, and a reported increased risk of bowel injury and pain [9].

In nonemergent settings, as in our case, the direct intercostal approach has been shown to be effective and safe [10, 27]. The application of prosthetic reinforcement is favored in most cases, especially for very large or recurrent defects [35], since the absence of prosthetic support is associated with higher rates of recurrence [21, 27]. For our patient, we opted to use an 8 cm diameter patch (VENTRALEX Hernia Patch) whose straps were anchored to the fascia of the external oblique and intercostal muscles. Some surgeons advocate the application of fibrin glue, instead of sutures or tacks, to anchor the mesh in an attempt to limit postoperative discomfort and mesh migration [9]. They report no hernia recurrence or discomfort at 2-year followup. Although these results are a reassuring alternative to sutures, more controlled studies are needed to determine the short term and long term clinical effectiveness of fibrin glue in AAIH repairs.

While Losanoff et al. found success using cable loops to approximate the ribs [2], such an approach, as a general rule, should be avoided as it may cause chronic pain and discomfort as well as intercostal nerve damage  [9, 31]. However, some authors advocate its use under special circumstances: when a displaced rib creates a widened intercostal space, when there is a very large defect, or when the periosteum of the ribs provides a more secure anchoring structure than the tissue around the defect, which in some patients may be weakened by scar tissue, comorbidities, or congenital syndromes that compromise tissue integrity [2, 4, 9, 13, 14, 24, 32–34]. In the preoperative planning for our patient, we decided that the use of cables was unnecessary, since there was no displaced or fractured rib to create a widened intercostal space; also, we wanted to avoid the risk of chronic pain symptoms in the patient.

Regardless of approach, the most recent comprehensive literature review on AAIH by Erdas et al. reports that recurrences occurred in 28.6% [9] of cases and were seen in up to 12 months [29]. This number could be underestimated, since several cases had short follow-up times of less than 3 months [24, 25, 30] or were not followed up at all [33, 34]. Theories explaining the high recurrence rate are missed diaphragmatic tear [2], ripping of sutures, or the development of another defect from the jagged edges of rib fractures [10, 35]. Future studies are needed to shed light on more effective ways to prevent recurrent hernias.

In conclusion, physicians must maintain a high index of suspicion for both abdominal and transdiaphragmatic intercostal hernias in patients who present with palpable bulges over the chest wall, especially in those with a history of penetrating or blunt trauma to the abdomen and thorax. The CT is the diagnostic instrument of choice. Because there are so few reports of acquired abdominal intercostal hernias, determining the efficacy of various surgical techniques employed is difficult. The surgeon's experience and patient factors should be considered before deciding on a repair technique. Although the rates of recurrences and complications for AAIH have limited statistical credence, the cases reported in the literature do lend support for their potential in causing significant morbidities. Therefore, swift surgical management should be pursued in symptomatic patients with AAIH.
